# Supplementary material for: Determination of Density Functional Tight Binding Models for Cerium-Containing Materials
Source: J Chem Theory Comput. 2026 Aug 19;22(17):8717–24. doi: 10.1021/acs.jctc.6c00892 (PMC13564360; doi:10.1021/acs.jctc.6c00892)
Supplement: Supplementary file 1 [file ct6c00892_si_001.pdf]

Supporting Information: Determination of Density Functional Tight Binding Models for Cerium-containing Materials

Nir Goldman,[*,†,‡] Artem Samtsevych,[¶] and Chiara Panosetti[¶]

†*Physical and Life Sciences Directorate, Lawrence Livermore National Laboratory, Livermore, CA 94550 USA*

‡*Department of Chemical Engineering, University of California, Davis, California 95616, United States*

¶*Fritz Haber Institute of the Max Planck Society, 14195 Berlin, Germany*

E-mail: ngoldman@llnl.gov

1 Modifications to DSKO

We have included new options in DSKO to allow for relativistic calculations for higher-Z elements (through the Zeroth-Order Regular Approximation) and the fraction of exact exchange for the hybrid functional. PBE0 calculations are performed using the truncated Coulomb matrix method with a cutoff of 20.0 au and neighbour-based screening with a threshold of 10^{-5}. These parameters were validated against α-Ce calculations using a cutoff of 40.0 au and a threshold of 10^{-5}. We enforce an odd k-point mesh, as required for many hybrid functional calculations in order to avoid singularities at the Γ-point. Shell resolved SCC is also turned off, as required for hybrid functionals by the current DFTB+ release.

2 ChIMES Methodology

An in-depth review of ChIMES can be found in Ref. 1. Briefly, in the ChIMES formalism, we represent each of the terms in our n-body energy expansion as a linear combination of Chebyshev polynomials. Chebyshev polynomials of the first kind of order m are defined by the expression $T_m(\cos\theta) = \cos(m\theta)$, more commonly written as $T_m(x)$, where $x = \cos\theta$ and thus exists over the range $[-1, 1]$. The derivatives of Chebyshev polynomials of the first kind are related to Chebyshev polynomials of the second kind $U_m(x)$ by the expression $\mathrm{d}T_m/\mathrm{d}x = mU_{m-1}$, where $U_m(\cos\theta) = \sin[(n+1)\theta]/\sin\theta$.

We write the two-body (2B) energy term as the following expression:

$$^2E_{i_1 i_2} = f_\mathrm{p}(r_{i_1 i_2}) + f_\mathrm{c}^{e_{i_1} e_{i_2}}(r_{i_1 i_2}) \sum_{m=1}^{\mathcal{O}_2} C_m^{e_{i_1} e_{i_2}} T_m(s_{i_1 i_2}^{e_{i_1} e_{i_2}}) \tag{1}$$

$C_m^{e_{i_1} e_{i_2}}$ is an optimized coefficient for the interaction between atom types e_{i_1} and e_{i_2}, taken from the set of all possible element types, $\{\boldsymbol{e}\}$. All $C_m^{e_{i_1} e_{i_2}}$ are permutationally invariant.

$T_m\left(s_{i_1 i_2}^{e_{i_1} e_{i_2}}\right)$ represents a Chebyshev polynomial of order m, and $s_{i_1 i_2}^{e_{i_1} e_{i_2}}$ is the pair distance transformed to occur over the interval $[-1, 1]$ using a Morse-like function.[2,3] For that coordinate transform, $s_{i_1 i_2}^{e_{i_1} e_{i_2}} \propto \exp\left(-r_{i_1 i_2}/\lambda_{e_1 e_2}\right)$ and $\lambda_{e_1 e_2}$ is an element-pair distance scaling constant, frequently set to the first peak in a radial distribution function. Further details are discussed in Ref. 4. The term $f_{\mathrm{c}}^{e_{i_1} e_{i_2}}(r_{i_1 i_2})$ is a Tersoff cutoff function[5] which smoothly varies to zero up to a predefined maximum cutoff distance. In order to prevent sampling of $r_{i_1 i_2}$ distances below those sampled in our training set, we introduce use of a smooth repulsive penalty function $f_{\mathrm{p}}(r_{i_1 i_2})$ that is non-zero for distances close to the inner cutoff of the Chebyshev polynomials.

Many-body (e.g., greater than two-body) orthogonal polynomials can be created by defining a cluster of size n and taking the product of the Chebyshev polynomials derived from the constituent $\binom{n}{2}$ unique pairs. For example, the three-body polynomials will be products of $\binom{3}{2} = 3$ two-body polynomials. We thus write the ChIMES three-body (3B) energy as the following:

$$^{3}E_{i_1 i_2 i_3} = f_{\mathrm{c}}^{e_{i_1} e_{i_2}}\left(r_{i_1 i_2}\right) f_{\mathrm{c}}^{e_{i_1} e_{i_3}}\left(r_{i_1 i_3}\right) f_{\mathrm{c}}^{e_{i_2} e_{i_3}}\left(r_{i_2 i_3}\right) \sum_{m=0}^{\mathcal{O}_3} \sum_{p=0}^{\mathcal{O}_3} \sum_{q=0}^{\mathcal{O}_3}{}' C_{mpq}^{e_{i_1} e_{i_2} e_{i_3}} T_m\left(s_{i_1 i_2}^{e_{i_1} e_{i_2}}\right) T_p\left(s_{i_1 i_3}^{e_{i_1} e_{i_3}}\right) T_q\left(s_{i_2 i_3}^{e_{i_2} e_{i_3}}\right). \tag{2}$$

We thus compute a triple sum for the product of the $i_1 i_2$, $i_1 i_3$, and $i_2 i_3$ pair-wise polynomials. These are computed up to a predefined order ($\mathcal{O}_3$) for each three-body polynomial and then multiplied by a single coefficient, $C_{mpq}^{e_{i_1} e_{i_2} e_{i_3}}$, that is permutationally invariant for each set of polynomial orders and atom types. The primed sum in Equation 2 indicates that only terms for which two or more of the m, p, q polynomial powers are greater than zero are included in order to guarantee that all atoms in the cluster interact through Chebyshev polynomials. The expression for $^{3}E_{i_1 i_2 i_3}$ also contains the f_{c} smoothly varying cutoff functions for each constituent pair distance. The penalty function f_p is not included here.

Higher bodied terms are included in ChIMES in a similar fashion. For example, four-body (4B) terms are often included in ChIMES optimizations,[6] where ${}^4E_{i_1i_2i_3i_4}$ is now determined from the sum over the product of the $\binom{4}{2} = 6$ constituent pair-wise polynomials multiplied by a single permutationally invariant coefficient. In the determination of permutational invariance for an arbitrary number of bodies, it is important to realize that the atom indices and atom types are permuted, which then implies a corresponding permutation of the bond distance indices in C.

Similar to other machine learning atomic interaction potentials, ChIMES uses the method of force matching[7] to determine the interaction parameters. In force matching a training set of quantum simulations is generated with differing configurations. For each configuration in the training set, the quantum mechanical energy, atomic force, and stress (for condensed phase configurations) are calculated. In addition, weights can be used when matching to forces, energies, and stresses, due to the differing physical units and number of parameters per configuration. In this work, we use a weight of $5/n_a$ for the energies (n_a is equal to the number of atoms in a give configuration), and a value of one for the forces. Stress tensor components have been omitted from these optimizations, though we include them in our discussion for the sake of clarity. Once weights are determined, an objective function for optimization may be defined as follows:

$$F_{\text{obj}} = \frac{1}{N_d} \sum_{\tau=1}^{M} \left(\sum_{i=1}^{N_\tau} \sum_{\alpha=1}^{3} \left(w_{\text{F}} \Delta \text{F}_{\tau_{\alpha_i}} \right)^2 + \sum_{\alpha=1}^{3} \sum_{\beta \leq \alpha} \left(w_\sigma \Delta \sigma_{\tau_{\alpha\beta}} \right)^2 + \left(w_{\text{E}} \Delta E_\tau \right)^2 \right). \tag{3}$$

Here, τ corresponds to a specific training set configuration, i is the atomic index, and α and β are the cartesian directions. M is the total number of configurations in the training set and $N_d = 3\sum_\tau N_\tau + 7M$ is the total number of data entries (6 stress tensor components and one energy value per configuration). In addition, $\Delta F_{\tau_{\alpha_i}} = F_{\tau_{\alpha_i}}^{\text{ChIMES}} - F_{\tau_{\alpha_i}}^{\text{DFT}}$, $\Delta\sigma_{\tau_{\alpha\beta}} = \sigma_{\tau_{\alpha\beta}}^{\text{ChIMES}} - \sigma_{\tau_{\alpha\beta}}^{\text{DFT}}$, and $\Delta E_\tau = E_\tau^{\text{ChIMES}} - E_\tau^{\text{DFT}}$. The value w_{F} is the weight for forces, w_σ for stresses, and w_{E} for energies.

Optimal ChIMES parameters are determined by solving

$$\frac{\partial F_{\text{obj}}}{\partial C_I} = 0, \tag{4}$$

for all I, where I is a combined index of the permutationally unique coefficient. Optimizing F is equivalent to solving the overdetermined matrix equation

$$\boldsymbol{wAC} = \boldsymbol{wB}. \tag{5}$$

The matrix $\boldsymbol{A}$ corresponds to the derivatives of the ChIMES energy, stress, or force expression with respect to the fitting coefficients. The column vectors $\boldsymbol{C}$ and $\boldsymbol{B}$ correspond to the ChIMES coefficients to be optimized and the numerical values for the training data, respectively. The diagonal matrix $\boldsymbol{w}$ is comprised of the weights to be applied to the elements of $\boldsymbol{B}$ and rows of $\boldsymbol{A}$. Solution to this linear least-squares problem can performed using a number of different optimization algorithms, discussed in Ref. 1.

3 ChIMES parameters and fitting results

For this each of the ChIMES repulsive energies discussed in this work, we have used a minimum radial cutoff of 2.35 Å where 2.40 Å was the minimum cerium nearest neighbor distance sampled in our training set. We have set the ChIMES. maximum cutoff to a value of 4.35 Å which ensures inclusion of second-nearest neighbor interactions for the molecular dynamics simulations discussed in the manuscript. Use of a cutoff that included nearest-neighbor interactions only (e.g., 3.40 Å) resulted in similar results for the energetic ordering of the cerium polymorphs but worse agreement with DFT for the vibrational density of states. Optimizations were performed with the LASSO/LARS algorithm, using a regularization parameter value of 1×10^{-4}, and included energy and force data, only.

Table S 1: Root mean squared (RMS) errors from our ChIMES repulsive energy optimizations.

ChIMES E_{Rep} bodiedness	Force (eV/Å)	Energy (eV/atom)
2B	0.21	0.06
3B	0.14	0.05
4B	0.14	0.05

4 DSKO optimized DFTB-PBE parameters

Table S 2: Woods-Saxon confinement parameters and Hubbard U values for Ce.

Shell	r_0	a	W	Hubbard U (eV)
4f	4.1068	13.5678	1.2146	0.4670
5d	4.5135	60.0000	6.0000	0.2247
6s	4.0472	60.0000	1.0000	0.1640
6p	5.9384	59.0451	1.3237	0.2247
Density	23.8488	5.0000	18.2151	—

Table S 3: PBE spin constant matrix for Ce (in eV).

	4f	5d	6s	6p
4f	-0.01235	-0.00245	-0.00078	-0.00245
5d	-0.00245	-0.00902	-0.00582	-0.00902
6s	-0.00078	-0.00582	-0.01009	-0.00582
6p	-0.00245	-0.00902	-0.00582	-0.00902

5 DSKO optimized DFTB-PBE0 parameters

Table S 4: Confinement parameters and Hubbard U values for Ce. Shell-resolved SCC is not available for hybrid functionals in the DFTB+ code,[8] hence the equal values for all U.

Shell	r_0	a	W	Hubbard U (eV)
4f	11.6330	25.3199	2.3849	0.3590
5d	5.8006	42.1194	4.1573	0.3590
6s	5.2377	38.0321	2.3187	0.3590
6p	3.9278	31.0841	1.1961	0.3590
Density	20.1927	34.5318	36.2038	—

Table S 5: PBE0 spin constant matrix for Ce (in eV). Identical results were obtained for for Ce and CeO_2 parameterizations since the STO basis set remained unchanged for these calculations.

	4f	5d	6s	6p
4f	-0.01885	-0.00281	-0.00082	-0.00281
5d	-0.00281	-0.01000	-0.00604	-0.01540
6s	-0.00082	-0.00604	-0.01249	-0.00604
6p	-0.00281	-0.01540	-0.00604	-0.00625

Table S 6: Decoding mask used in CeO_2 DSKO optimizations. All band positions are shown relative to the uppermost valence band, which is defined as '0'.

Special k-point	Bands
Γ	$[-3, -2, -1, 0, 1, 4]$
X	$[-3, -2, -1, 0, 1]$
W	$[-3, -2, -1, 0, 1]$
K	$[-3, -2, -1, 0, 1]$
L	$[-3, -2, -1, 0, 1]$
U	$[-3, -2, -1, 0, 1]$

Table S 7: Confinement parameters and Hubbard U values for Ce and O used in the Ce–O parametrization.

Element	Shell	r_0	a	W	Hubbard U (eV)
Ce	4f	4.0056	30.3612	5.2896	0.1645
Ce	5d	9.5332	35.5609	3.9580	0.1645
Ce	6s	7.6182	41.2194	3.5980	0.1645
Ce	6p	7.4353	35.2704	2.3549	0.1645
Ce	Density	17.1335	43.4927	47.9009	—
O	2s	5.1296	25.5178	3.5042	0.5168
O	2p	6.5569	49.4811	4.4037	0.5168
O	Density	16.0555	82.3198	20.9091	—

Table S 8: PBE0 spin constant matrix for O used in the Ce–O parametrization (in eV).

	2s	2p
2s	-0.06540	-0.04334
2p	-0.04334	-0.03796

6 CeO_2 band structure orbital projections

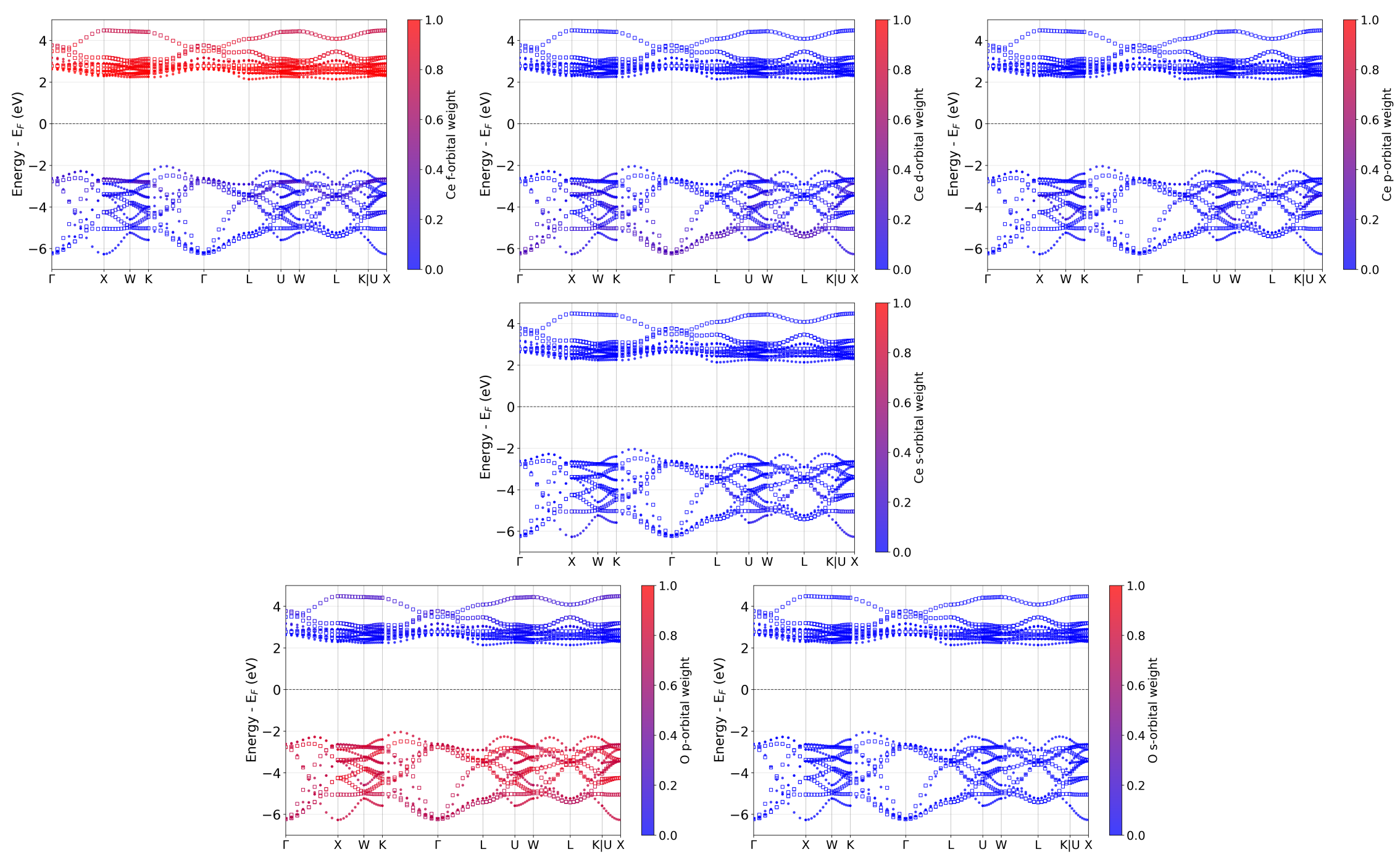

Figure S 1: Atom resolved band structure projections for CeO_2 from our DFTB-PBE0 model. Closed circles correspond to results from DFT and open squares to DFTB+. Red indicates increased orbital projection values.

References

(1) Goldman, N.; Fried, L. E.; Lindsey, R. K.; Pham, C. H.; Dettori, R. Enhancing the accuracy of density functional tight binding models through ChIMES many-body interaction potentials. *J. Chem. Phys.* **2023**, *158*, 144112.

(2) Wang, Y.; Shepler, B. C.; Braams, B. J.; Bowman, J. M. Full-dimensional, ab initio potential energy and dipole moment surfaces for water. *J. Chem. Phys.* **2009**, *131*, 054511.

(3) Wang, Y.; Huang, X.; Shepler, B. C.; Braams, B. J.; Bowman, J. M. Flexible, ab initio potential, and dipole moment surfaces for water. I. Tests and applications for clusters up to the 22-mer. *J. Chem. Phys.* **2011**, *134*, 094509.

(4) Lindsey, R. K.; Fried, L. E.; Goldman, N. ChIMES: A Force Matched Potential with Explicit Three-Body Interactions for Molten Carbon. *J. Chem. Theory Comput.* **2017**, *13*, 6222–6229.

(5) Tersoff, J. Empirical interatomic potential for carbon, with application to amorphous-carbon. *Phys. Rev. Lett.* **1988**, *61*, 2879.

(6) Pham, C. H.; Lindsey, R. K.; Fried, L. E.; Goldman, N. Calculation of the detonation state of HN_3 with quantum accuracy. *J. Chem. Phys.* **2021**, *153*, 224102.

(7) Ercolessi, F.; Adams, J. B. Interatomic potentials from first-principles calculations: The force-matching method. *Europhys. Lett.* **1994**, *26*, 583–588.

(8) Hourahine, B.; Berdakin, M.; Bich, J. A.; Bonafé, F. P.; Camacho, C.; Cui, Q.; Deshaye, M. Y.; Díaz Mirón, G.; Ehlert, S.; Elstner, M.; Frauenheim, T.; Goldman, N.; González León, R. A.; van der Heide, T.; Irle, S.; Kowalczyk, T.; Kubař, T.; Lee, I. S.; Lien-Medrano, C. R.; Maryewski, A.; Melson, T.; Min, S. K.; Niehaus, T.; Niklasson, A. M. N.; Pecchia, A.; Reuter, K.; Sánchez, C. G.; Scheurer, C.; Sentef, M. A.;

Stishenko, P. V.; Vuong, V. Q.; Aradi, B. Recent Developments in DFTB+, a Software Package for Efficient Atomistic Quantum Mechanical Simulations. *The Journal of Physical Chemistry A* **2025**, *129*, 5373–5390.
